# Cervicothoracic Arachnoid Cyst Causing Cervical Myelopathy: A Case Report

**DOI:** 10.3889/oamjms.2015.026

**Published:** 2015-02-13

**Authors:** Zahir Kizilay, Ali Yilmaz, Ayca Ozkul, Ozgur Ismailoglu

**Affiliations:** 1 *Adnan Menderes University, Medical Faculty, Neurosurgery, Aytepe Campus, Aydin 09100, Turkey*; 2 *Adnan Menderes University, Medical Faculty, Department of Neurology, Aydin 09100, Turkey*; 3 *Süleyman Demirel University, Neurosurgery, 32260 Isparta, Turkey*

**Keywords:** Cervical spondylosis, anterior subarachnoid space, cerebrospinal fluid, arachnoid cyst, myelopathy

## Abstract

Several types of intraspinal cyst develop within the spinal canal from the craniovertebral junction to the sacrum. These lesions occur in both children and adults. Arachnoid cysts are one of them and are more frequent in the paediatric population, being a relatively uncommon lesion in adults. The arachnoid cyst may be located intradurally or extradurally. The intradural type may be congenital or from spinal trauma, infection or spondylosis. Although intradural arachnoid cysts are often asymptomatic, they may give early symptoms when they exist with synchronous pathologies constricting the spinal canal gradually as in cervical spondylosis. In this report, a 60-year-old man with an arachnoid cyst of the cervicothoracic spine is presented. His cyst remained undiagnosed because of the nonspecific nature of the symptoms. It was only when he developed right hemiparesis that a posterior fluid collection compressing the spinal cord was found in Magnetic resonance imaginig. An intradural extramedullary cyst was removed with successful surgery and cord compression and symptoms were reversed. We discuss radiological diagnosis and surgical treatment of an arachnoid cyst in this report.

## Introduction

Many intradural or extradural pathologies extending from the cervical region to the thoracic region and compressing the cervical spinal cord can lead to cervical myelopathy. Arachnoid cysts are mostly intradural and are rarely extradural entities. Their reported localisations are often the thoracic and cervical regions as well as rarely the lumbar region [1-4]. They are usually found incidentally and do not require surgical operation. Especially in cervical spondylosis, which develops with advanced age, the spinal canal may become narrowed and primarily harmless structures of the spinal canal such as arachnoid cysts may cause constriction in the spinal canal and neurological deterioration may occur [1, 5, 6]. This case report examines a patient who received posterior decompression and fixation treatment, who had an arachnoid cyst extending from the cervical region to upper the thoracic region and cervical spondylosis and resultant cervical myelopathy.

## Case presentation

The patient was a 60 year old male who had intermittent numbness in his right arm and leg. The numbness had started a year ago for the first time. Approximately six months later he had a pain in his right arm. He did not benefit from medication given to him and he had further complaints. First, a weakness developed in his right arm and approximately three months later the weakness spread to his right leg.

One month after the development of evident hemiparesis, he presented to our clinic with complaints of weakness and numbness in his left leg. His physical examination revealed: muscles strength in the right upper extremity of 2/5, in the right lower extremity 3/5, in the left upper and lower extremity 4/5, below C5 bilateral hemihypoesthesia prominent at right, hyperactive deep tendon reflexes in bilateral upper and lower extremities and positive Babinski sign in bilateral lower extremities. Urinary and faecal incontinence were not present. Cervical and lumbar magnetic resonance imaging (MRI) were performed.

**Figure 1:**
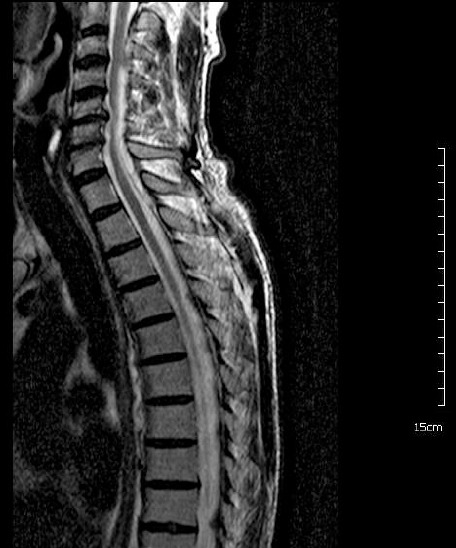
*A cystic mass lesion which was extends between C3 to T2 in preoperative T2 MRI sagittal section*.

**Figure 2:**
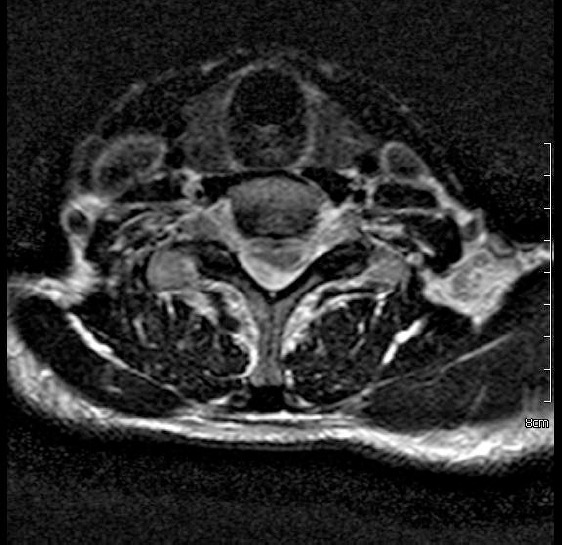
*A cystic mass is located right posterolaterally to the spinal cord in preoperative T2 MRI axial section*.

Cervical MRI revealed a non contrast cystic mass lesion posterolaterally located on the right and extending between C3-T2 and an increase in signal density between C5-6 levels of the spinal cord compatible with myelomalacia in T2 MRI weighted sequence (Fig. 1, 2).

**Figure 3:**
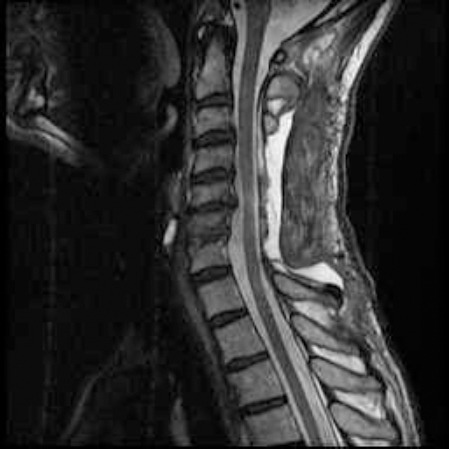
*There is no arachnoid cyst and the spinal cord anterior subarachnoid space has expanded in postoperative T2 cervical MRI sagittal section*.

**Figure 4:**
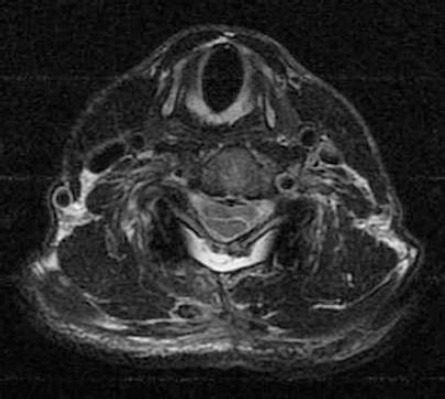
*There is no arachnoid cyst in postoperative T2 MRI axial section*.

The patient underwent laminectomy from C4 to the upper edge of C7. The dura was medially incised and thus a thin walled arachnoid cyst was seen (located onthe right) which was pushing the spinal cord leftward. The integrity of the cyst was destroyed via bipolar. The dura was repaired with primary suture. Lateral mass screwing was performed between the C3 and C7 levels. Post-operative patient follow-ups showed that the upper and lower extremities were relieved and muscle strength was increased. The patient was transferred to another healthcare centre in order to receive physiotherapy. 45 days later, checkup MRI was performed. This revealed spinal cord expansion and that the cyst was not present (Fig. 3, 4).

In the most recent physical examination muscle strength in each of the patient’s four extremities was found to be totally normal.

## Discussion

Spinal arachnoid cysts are also called dural cysts and they originate from the arachnoid membrane of the spinal cord. They may be in an extradural or an intradural localisation. Arachnoid cysts occur for secondary reasons which cause arachnoid adhesions such as congenital causes, tumour, trauma and infection [2, 7-10]. Arachnoid cysts have been divided into three groups by researchers: type 1, with extradural localisation and not containing nerve root: type 2, with extradural localisation and nerve root contact: and type 3, arachnoid cysts with intradural localisation and often located in posterior and posterolateral parts [11, 12]. Our case can be regarded as a type 3 arachnoid cyst because of its both intradural and posterolateral localisations. Generally, type 3 arachnoid cysts can occur in any place throughout the posterior spinal subarachnoid space. Intradural cysts occur particularly in the thoracic region but they can also occur in the cervical (15%) and lumbar regions (5%) [3, 13]. Intradural cysts are often asymptomatic and multiple [3, 17]. Findings and symptoms can vary depending on the localisation of the cysts when the cysts become symptomatic. While cysts on the ventral surface often cause weakness and myelopathy, cysts on the dorsal side primarily cause neuropathic pain and numbness. These differences changes in symptoms can be explained with local compression of spinal tracts by cysts or alternatively by vascular compression of the anterior spinal artery by cysts which are located on the ventral surface, which may cause symptoms such as myelopathy and weakness [7].

Advanced age related acquired quadriparesis is the most common reason for cervical spondylotic myelopathy. Cervical spondylotic myelopathy is a clinical condition resulting from constriction and insufficient nutrition dependent on damage which occurs in white and gray matter as a result of solitary or combined pathologies of the anterior, medial and posterior spinal cord such as posterior longitudinal ligament ossification, disc degeneration, facet joint hypertrophy or ligamentum flavum hypertrophy [2, 14-16]. Spondylosis is shown to be an etiological factor, especially for the development of arachnoid cysts. Therefore congenital cysts or arachnoid cysts secondary to spondylosis can exist synchronously with cervical spondylosis [2].

Although intradural arachnoid cysts are often asymptomatic, they may give early symptoms when they exist with synchronous pathologies constricting the spinal canal gradually such as cervical spondylosis and these early symptoms appearing in correlation with the severity of cervical spondylosis. This is because in patients whose anterior subarachnoid compartment isconstricted in different parts, the volume of cerebrospinal fluid (CSF) entering the cysts may be increased due to the degree of compression applied on the ventral surface, but during the asymptomatic period of arachnoid cysts which are often found in a posterior or posterolateral location, CSF can circulate through the cyst. Thus, constriction of the canal secondary to spondylosis, in addition to increased spinal cord compression due to the mass effect of an arachnoid cyst located in the CSF can induce clinical symptoms and the patient’s condition can deteriorate. This does not contradict the literature, because when the secondary reasons for intradural cysts are searched in the literature, many reasons such as history of trauma, surgery, infections causing meningitis and spinal subarachnoid haemorrahage are reported. However, with the knowledge that most intradural arachnoid cysts are congenital and asymptomatic, it can be predicted that congenital arachnoid cysts can become symptomatic with secondary causes which constrict the spinal cord, such as spondylosis. Onset and progress of the symptoms and development of cervical myopathy symptoms takes approximately one year as happened in our case. When the long development process of spondylosis is considered, one year is a possible time period for the development of clinical symptoms in patients with a posterolateral arachnoid cyst. In the literature, despite there being 11 intramedullary arachnoid cyst cases and one cervical spondylosis case together with one intramedullary cyst case, there are no cases of cervical spondylosis together with intradural extramedullary arachnoid cyst other than ours [5].

Surgical treatment of cervical spondylotic myelopathy is still controversial. Some groups support an anterior approach while some other groups believe in the sufficiency of posterior decompression [18-21]. When the literature is examined, it can be understood that the posterior approach is often preferred for arachnoid cysts [1, 3-5, 7, 8, 12, 13]. Instead of surgery, clinical follow-up is suggested for asymptomatic arachnoid cysts [3, 12]. The posterior approach can be preferred for both posterior and posterolateral arachnoid cysts which cause clinical symptoms and pathologies affecting the spinal cord such as cervical spondylosis. We also preferred the posterior approach for the treatment of our patient who was diagnosed with both cervical spondylotic myelopathy and an arachnoid cyst. We detected improvements in the radiological and clinical parameters of the patient during his hospital follow–ups. Our self criticism here is whether we could have obtained the same degree of radiological and clinic improvements if we had performed posterior decompression without opening the dura. Although there is not certain answer to this question, arachnoiditis may occur in pathologies developing in the long term, such as spondylosis due to surrounding compression. It is obvious that decompression will not be sufficient for such cases. Furthermore, there is no data in the literature showing the sufficiency of posterior decompression in patients with synchronous cervical spondylotic myelopathy and arachnoid cyst. Also, no data exists about which treatment has better outcomes.

To conclude, arachnoid cysts should be treated surgically when they are seen synchronously with pathologies affecting the spinal cord such as cervical spondylosis, because this condition may cause early clinical symptoms and progressive neurological deficits. Performing spinal MRI before surgery enables the diagnosis of intradural cysts and helps to plan surgery according to the anterior or posterior location of arachnoid cysts.
